# Moderate intensity continuous versus high intensity interval training: Metabolic responses of slow and fast skeletal muscles in rat

**DOI:** 10.1371/journal.pone.0292225

**Published:** 2023-10-04

**Authors:** Morgane Pengam, Christelle Goanvec, Christine Moisan, Bernard Simon, Gaëlle Albacète, Annie Féray, Anthony Guernec, Aline Amérand

**Affiliations:** EA 4324 ORPHY, Université de Brest, Brest, France; Gyeongsang National University, REPUBLIC OF KOREA

## Abstract

The healthy benefits of regular physical exercise are mainly mediated by the stimulation of oxidative and antioxidant capacities in skeletal muscle. Our understanding of the cellular and molecular responses involved in these processes remain often uncomplete particularly regarding muscle typology. The main aim of the present study was to compare the effects of two types of exercise training protocol: a moderate-intensity continuous training (MICT) and a high-intensity interval training (HIIT) on metabolic processes in two muscles with different typologies: *soleus* and *extensor digitorum longus* (EDL). Training effects in male Wistar rats were studied from whole organism level (maximal aerobic speed, morphometric and systemic parameters) to muscle level (transcripts, protein contents and enzymatic activities involved in antioxidant defences, aerobic and anaerobic metabolisms). Wistar rats were randomly divided into three groups: untrained (UNTR), *n* = 7; MICT, *n* = 8; and HIIT, *n* = 8. Rats of the MICT and HIIT groups ran five times a week for six weeks at moderate and high intensity, respectively. HIIT improved more than MICT the endurance performance (a trend to increased maximal aerobic speed, p = 0.07) and oxidative capacities in both muscles, as determined through protein and transcript assays (AMPK–PGC-1α signalling pathway, antioxidant defences, mitochondrial functioning and dynamics). Whatever the training protocol, the genes involved in these processes were largely more significantly upregulated in *soleus* (slow-twitch fibres) than in EDL (fast-twitch fibres). Solely on the basis of the transcript changes, we conclude that the training protocols tested here lead to specific muscular responses.

## Introduction

Regular practice of a physical activity is recognized to induce beneficial health effects by decreasing risk factors associated with metabolic diseases (such as cardiovascular diseases, type 2 diabetes, metabolic syndrome or cancers) or their progression [1, 2]. In these diseases, metabolic impairments such as decreased oxidative capacity and mitochondrial dysfunction often occur in skeletal muscle [3, 4].

Although the health benefits of regular exercise are now well documented, a proportion of the population remains inactive due to lack of time and/or motivation. High-intensity interval training (HIIT) provides a way of getting some benefits (improvement of maximal oxygen consumption ($\dot{V}O_{2}\max$), muscle oxidative capacity, insulin sensitivity) of moderate-intensity continuous training (MICT) while spending less time doing physical exercise [2, 5, 6]. HIIT, alternating periods of high and low intensity exercise, also offers the advantage of improving anaerobic capacity [7]. In human and rodents, HIIT could be more effective than MICT in the improvement of oxidative capacities to stimulate mitochondrial biogenesis and/or oxidative phosphorylation (OXPHOS) in skeletal muscle [8, 9]. HIIT and MICT can also stimulate antioxidant defences but sometimes do this differently according to the training intensity or to the tissue considered [10, 11]. Our understanding of the cellular and molecular mechanisms underlying these beneficial training effects is often still incomplete, particularly regarding muscle typology. Indeed, the skeletal muscles differ in their fibre composition (slow-twitch or fast-twitch fibres, which are respectively rich and poor in mitochondria). The fibre type-dependent composition should also be correlated with muscle performance and muscle-associated metabolic diseases [12, 13]. Another recent study has shown that the expression of numerous proteins involved in the mitochondrial metabolism also adapts to training in a fibre type-specific manner [14].

In human and in rodents, the signalling pathway AMPK–PGC-1α is one of the main pathways that increases oxidative capacities in response to regular physical exercise [15, 16]. AMPK upregulates PGC-1α, which in turn stimulates the expression of mitochondrial and antioxidant genes [17]. Among its numerous functions, PGC-1α could regulate the dynamics of mitochondrial fission and fusion [18]. PGC-1α could also drive a change in fibre type from fast- to slow-twitch fibre [19]. The molecular network underlying these processes involved in the improvement of oxidative capacity with MICT and HIIT is still only partially elucidated.

To our knowledge, the training effects on mitochondrial biogenesis and antioxidant processes have usually been studied separately in relation to the type of muscle and/or exercise [20, 21]. Moreover, results often vary from one study to another [22, 23]. Here, the main purpose is to determine, in a same study, the effects of two common training protocols (MICT and HIIT) on aerobic and anaerobic responses in healthy Wistar rats in two muscles with different typologies over six weeks. A murine model was preferentially chosen to explore these molecular mechanisms because comparative future investigations on skeletal muscles and cardiovascular system will be made using a rat model of metabolic syndrome. Different measurements were explored at the whole animal level: maximal aerobic speed (MAS), classical morphometric and cardiovascular parameters (heart rate, arterial blood pressure and cutaneous microvascular endothelial function). Mitochondrial and antioxidant enzyme activities as well as gene expression and transcription (AMPK–PGC-1α, mitochondrial functioning and dynamics, antioxidant enzymes, lactate dehydrogenase and myosin heavy chain) were measured in *soleus* and *extensor digitorum longus* (EDL), which are mainly oxidative and glycolytic, respectively. It is known that the recruitment of fibres in each muscle depends on the intensity and the duration of exercise [24, 25]. Submaximal work is performed by the more aerobically efficient slow-twitch fibres while progressively increasing numbers of fast-twitch fibres are recruited to assist them as the effort increases toward the maximum [7]. Because of the different typology of *soleus* and EDL, we hypothesized that the two training protocols MICT and HIIT could induce different metabolic adaptations in each of them.

## Materials and methods

### Animals

Twenty-three male Wistar rats (21 days, 52.1 ± 0.6 g, Janvier Labs, Le Genest Saint Ile, France), all born on the same day, were housed at least two per cage in a light (12h:12h light/dark cycle) and temperature (21 ± 1°C) controlled animal facility until the age of 15 weeks. The rats had access to a standard chow diet (KLIBA NAFAG®, Kaiseraugst, Germany, Mouse and Rat Maintenance, 3.152 kcal/g) and drinking water *ad libitum*. Body weight, food, drink and total calorie intakes per rat were measured individually once a week. Weight gain and total calorie intake were calculated for the whole training period. All experiments were approved by the *Comité d’Éthique Finistérien en Expérimentation Animale* n°74 and authorized in writting by the French *Ministère de l’Éducation Nationale*, *de l’Enseignement Supérieur et de la Recherche* (APAFIS#17956-2018120517015356v4).

### Familiarization with the treadmill and test of maximal aerobic speed

At 8 weeks of age, all rats followed a treadmill familiarization protocol during four consecutive days. Daily session duration was gradually increased over this period from 30 min to 45 min of running at speeds of 8.3 to 20.0 m/min. At the end of this week, the maximal aerobic speed (MAS) of each rat was determined. The MAS test protocol consisted of an exercise session where the starting speed of 10 m/min was progressively incremented every 60 s by 3.33 m/min until reaching 26.7 m/min, and then by 1.7 m/min until rats were unable to run anymore [1]. The last speed fully completed was taken as their MAS. At 9 weeks of age, the rats were randomly assigned to one of three groups: untrained (UNTR, *n* = 7), moderate-intensity continuous training (MICT, *n* = 8) or high-intensity interval training (HIIT, *n* = 8). Only for MICT and HIIT groups, MAS was re-evaluated at the end of the third and last training week to adapt training intensity and evaluate exercise efficiency, respectively.

### MICT and HIIT protocols

MICT consisted of a 10-min warm-up to 33–49% of the rat’s MAS, followed by 50 min of running at 65% of their MAS. The training ended with an active recovery of 3 min at 20–30% of their MAS, giving a total of 63 min of exercise. HIIT began with a progressive 10-min warm-up to progressively reach approximately 70% of their MAS, followed by 5 cycles of 5 min consisting of: 2 min at 85–90% of their MAS followed by 3 min of active recovery at 30% of their MAS, totalling 35 min of exercise. Both trainings lasted six consecutive weeks (five times per week in the morning, with two consecutive days of rest during the weekend). The UNTR group was also brought to the running room each day so that they underwent the same transport conditions (from the animal facility to the laboratory running room) and these rats were also put on the switched-off treadmill.

### Arterial blood pressure and heart rate measurements

Arterial blood pressure (mean: MBP, systolic: SBP, and diastolic: DBP) and heart rate were determined by a non-invasive method that measures these parameters in the tail of conscious rats using volume pressure recording sensor technology (CODA® non-invasive blood pressure system, Kent Scientific, USA). All rats were conditioned to the procedure over one week before data collection. Before making these measurements, the rats were placed in a retraining box and preheated to more than 32°C on a specific platform to dilate the tail arteries. At least ten consecutive pressure measurements were needed to obtain representative values of SBP and DBP for each rat.

### Laser doppler flowmetry

To assess the cutaneous microvascular endothelial function, we performed iontophoresis with pharmacological agents (acetylcholine: ACh and sodium nitroprussiate: SNP) coupled with laser doppler flowmetry (LDF). This method was previously described in Lambrechts *et al*. (2013) [26]. During the experimental procedure, the rats were continuously anesthetized with 2% isoflurane through a nose cone (TEM Sega, Pessac, France) and their corporal temperature was maintained at 37°C. Briefly, the cutaneous blood flow response to iontophoresis was assessed using a LD probe (Periflux PF 384; Perimed, Järfälla, Sweden) in the previously shaved thigh. Cutaneous LDF is measured through a multifibre laser probe (780 nm) around which is placed an iontophoretic sponge connected to a distribution electrode (Periflux PF 383; Perimed) and a dispersive electrode (Periflux PF 384; Perimed) placed on the rat’s paw. For endothelium-dependent and -independent vasodilation analysis, we measured blood flow changes in response to 1% ACh chloride solution (right thigh) and then to 1% SNP (left thigh), respectively, delivered through the skin using a low electrical current. Cutaneous blood flow was indexed, as was cutaneous vascular conductance (CVC), which was calculated as LD flux. Responses to ACh and SNP were presented as the percentage of CVC variation between baseline and iontophoretic response. The LDF signal intensity depends on velocity and concentration of moving blood cells in the site under examination.

### Sampling

Between 48 and 72 hours after the end of the training period, the rats were anesthetised with ketamine (Ketamine 100, Virbac, 80 mg/kg) and xylazine (Rompun 2%, Bayer, 12 mg/kg) injected intraperitoneally. Morphometric measurements were then performed: body weight, naso-anal body length, abdominal and thoracic circumferences. Blood was collected intraventricularly into 2 mL sampling tubes (pre-coated with EDTA 5%) and hematocrit was evaluated. Plasma was obtained after centrifugation for 5 min at 3000 *g* at room temperature, frozen in liquid nitrogen and then stored at −80°C. The rats were sacrificed by cervical dislocation. Adipose tissue (epididymal, omental-retroperitoneal-peritoneal, subcutaneous and total adipose tissue) was weighed before freezing. Right and left *soleus* and *extensor digitorum longus* (EDL) muscles were immediately frozen in liquid nitrogen and then stored at −80°C for later analysis.

Certain morphometric indices make it possible to characterize obesity [27, 28]:

■ $\mathrm{Body}\;\mathrm{mass}\;\mathrm{index}\;(\mathrm{BMI},{g/\mathrm{cm}}^{2})=\frac{\mathrm{Body}\;\mathrm{weight}\;(g)}{\mathrm{Naso}‐\mathrm{anal}\;\mathrm{length}\;(\mathrm{cm})}$
■ Circumference index = $\frac{\mathrm{Abdominal}\;\mathrm{circumference}}{\mathrm{Thoracic}\;\mathrm{circumference}}$
■ $\mathrm{Lee}\;\mathrm{index}=\frac{\sqrt[{3}]{\mathrm{Body}\;\mathrm{weight}\;(g)}}{\mathrm{Naso}‐\mathrm{anal}\;\mathrm{length}\;(\mathrm{cm})}\times 10$
■ $\mathrm{Adiposity}\;\mathrm{index}=\frac{\mathrm{Total}\;\mathrm{adiposity}\;\mathrm{mass}}{\mathrm{Body}\;\mathrm{weight}}\times 100$

### RNA extraction, reverse transcription and real-time reverse transcriptase-PCR (RT-PCR)

For each muscle type (*soleus* and EDL), right and left muscles were ground together in liquid nitrogen to obtain a homogeneous tissue powder. Total RNA was isolated from 30 mg of frozen muscles using the NucleoSpin^®^ RNA Set for NucleoZOL (Macherey Nagel, Hoerdt, France) and stored at −80°C as previously described in [29]. RNA concentrations were measured with a SimpliNano^TM^ spectrophotometer (Biochrom Spectrophotometers, Fisher Scientific, Illkirch, France). Their purity and their integrity were also checked.

Each sample RNA was reverse transcribed with the qScript^TM^ cDNA synthesis kit (Quanta BioSciences, VWR, Fontenay-sous-Bois, France) containing a reaction mix and reverse transcriptase. Obtained cDNA was diluted 10-fold for PCR experiments and stored at −20°C.

Real-time RT-PCR was realized with a 7500 Fast Real-Time PCR system (Applied Biosystems, Thermo Fisher Scientific, Illkirch, France) as previously described in Pengam *et al*. [29]. Briefly, target genes were amplified and quantified by SYBR^®^Green incorporation (EurobioGreen^®^ Mix qPCR 2x Lo-Rox; Eurobio Ingen, Courtaboeuf, France) with the specific primers presented in **Table 1**. The cycling conditions consisted in a denaturing step at 95°C for 2 min, followed by 40 to 50 cycles of amplification (denaturation: 95°C for 5 s; annealing/extension: 60°C for 30 s). A seven-point standard curve was used to determine the PCR efficiency of each primer pair (between 80% and 100%) and the transcript level of the different genes in all samples. Each gene was amplified in a single run, from triplicates for standard points and duplicates for sample points. Quantification was normalized using *actin β* mRNA, considered as a reference gene. This choice was validated by the absence of significant differences in *actin β* mRNA levels between experimental groups for each muscle (*soleus* and EDL; p > 0.05). All mRNA levels were first calculated with the ratio: $\frac{\mathrm{target}\;\mathrm{gene}\;\mathrm{mRNA}}{\mathrm{actin}\;\beta \;\mathrm{mRNA}}$ and expressed as fold change compared with UNTR group, which was set at 1.

**Table 1:** Primer sequences used for real-time RT-PCR analysis.

Target gene	Abbreviation	Primer sequences (5’ to 3’)	Data base	Accession number
Actin *β*	*Actin β*	(F) CTACAATGAGCTGCGTGTGG	GenBank	NM_031144.3
		(R) GGATGGCTACGTACATGGCT		
Adenosine monophosphate kinase α1	*Ampkα1*	(F) GAAGCATATGCTGCAGGTAGA	GenBank	NM_019142.2
		(R) CGGGTCTTCAGGAAAGAGATA		
Peroxisome proliferator-activated receptor-γ coactivator-1α	*Pgc-1α*	(F) ACCCACAGGATCAGAACAAAC	GenBank	NM_031347.1
		(R) TACTTGAGAAGCTCTGAGCAG		
Nuclear respiratory factor 1	*Nrf1*	(F) TCACAGACAGTAGTACAGACC	GenBank	NM_001100708.1
		(R) TTGGCAGTTCTGAAGCATCAG		
Nuclear respiratory factor 2	*Nrf2*	(F) TTAAGCAGCATACAGCAGGAC	GenBank	NM_031789.2
		(R) ATTGCTGTCCATCTCTGTCAG		
Citrate synthase	*Cs*	(F) GCAGAGGGAATAAACCGAACT	GenBank	AF461496.1
		(R) GGTACAGATTCCGGTAGATCT		
NADH dehydrogenase 1	*Nd1*	(F) CGCCTGACCAATAGCCATAAT	GenBank	NC_001665.2
		(R) TTCGACGTTAAAGCCTGAGAC		
Cytochrome *c* oxidase 2	*Cox2*	(F) AATCTCATCCGAAGACGTCCT	GenBank	NC_001665.2
		(R) GTCACTGTAGCTTGGTTTAGG		
Cytochrome *c* oxidase 4	*Cox4*	(F) CCTGAAGGAGAAGGAGAAGG	GenBank	NM_017202.1
		(R) ACTCATTGGTGCCCTTGTTCA		
Adenosine triphosphate synthase 6	*Atp synthase 6*	(F) CCTATGAGCAGGAGCCGTAA	GenBank	NC_001665.2
		(R) TGGGAATTAGGGAGATGGGG		
Fission protein 1	*Fis1*	(F) ACGCCTGCCGTTACTTCTTC	GenBank	XM_006249122.3
		(R) GCAACCCTGCAATCCTTCAC		
Optic atrophy protein 1	*Opa1*	(F) GGCACTTCAAGGTCGTCTCA	GenBank	NM_133585.3
		(R) CACTGCTCTTGGGTCCGATT		
Mitofusin 1	*Mfn1*	(F) ATCTGGTGGAGATACAGGGCT	GenBank	NM_138976.1
		(R) TCCCACAGCATTGCGTTGAT		
Mitofusin 2	*Mfn2*	(F) GCTCAGTCGGTTGGAAGTCA	GenBank	NM_130894.4
		(R) GAAAGGAGTGCCTGCCTGAT		
Dynamin-related protein 1	*Drp1*	(F) AGGTTGCCCGTGACAAATGA	GenBank	NM_053655.3
		(R) CACAGGCATCAGCAAAGTCG		
Superoxide dismutase 1	*Sod1*	(F) ATTAACTGAAGGCGAGCATGG	GenBank	NM_017050.1
		(R) TCCAACATGCCTCTCTTCATC		
Superoxide dismutase 2	*Sod2*	(F) TGGCTTGGCTTCAATAAGGAG	GenBank	NM_017051.2
		(R) AAGATAGTAAGCGTGCTCCCA		
Glutathione peroxidase 1	*Gpx1*	(F) TGCAATCAGTTCGGACATCAG	GenBank	NM_030826.4
		(R) TTCACCTCGCACTTCTCAAAC		
Catalase	*Cat*	(F) CATGAATGGCTATGGCTCACA	GenBank	NM_012520.2
		(R) AAGTCTTCCTGCCTCTTCAAC		
Myosin heavy chain I (H7)	*I*	(F) ACCTGATGGTGGATGTGGAG	GenBank	NM_017240.2
		(R) CTTCTGCTTCCACTCAACCA		
Myosin heavy chain IIa (H2)	*IIa*	(F) TGACAACTCCTCTCGCTTTGG	GenBank	NM_001135157.1
		(R) TTAAGCTGGAAAGTGACCCGG		
Myosin heavy chain IIx (H4)	*IIx*	(F) CAGAAGCGCAATGTTGAAGCT	GenBank	NM_001135158.1
		(R) TGAGAACGTTCTTGCGGTCTT		
Myosin heavy chain IIb (H1)	*IIb* ^*1*^	(F) TGGCAATGCCAAGACTGTGAG	GenBank	NM_019325.1
		(R) AGGTTTCGATATCTGCGGAGG		
Lactate dehydrogenase A	*Ldh-a*	(F) TCATCCACTGAGCTGTCACG	GenBank	NM_017025.1
		(R) TGCTCCTTGTCTGCATCCGT		
Lactate dehydrogenase B	*Ldh-b*	(F) CAGGAACTGAACCCAGAGATG	GenBank	NM_001316333.1
		(R) CCCAGTTGGTGTAGCCTTTTA		

The hybridization temperature was 62°C for all primers. We designed all primers except those with a superscript number. (F): Forward, (R): Reverse. Reference: ^1^Hashimoto *et al*. (2016) [30]

### Western blot

Samples of 30 mg of frozen *soleus* or EDL were homogenized in 500 μL of RIPA buffer (10 mM Tris-HCl pH 7.5, 100 mM NaCl, 1 mM EDTA, 1% v/v deoxycholate sodium, 1% v/v Triton X100, 1% v/v Igepal, 0.1% v/v SDS 20%, 1% v/v protease and phosphatase inhibitor cocktail and 10 mM fluoride sodium).

The total protein concentration was measured in each muscle sample in a 96-well plate using BCA Protein Assay (Thermo Fisher Scientific). Briefly, samples were diluted 10 times and 200 μL of BC Assay reagent were added to 25 μL of diluted samples. Absorbance was read at 562 nm and total protein concentration calculated using a bovine serum albumin (BSA) standard range.

Samples were diluted in Laemmli Buffer 5X (10 mM Tris-HCl pH 6.8, 1% v/v SDS 20%, 25 mM EDTA pH 7.5, 8% v/v glycerol and 0.001% v/v bromophenol blue). From each sample 15 μg of proteins were run on 8–16% SDS-polyacrylamide gels, and proteins were then semi-dry transferred to a 0.2 μm PVDF membrane (BioRad). The membrane was washed 3 times in TBST (25 mM Tris pH 7.5, 150 mM NaCl and 1% v/v Tween 20) for 10 min. The membrane was then cut into three parts: (1) 10 to 50 kDa (containing GAPDH), (2) 50 to 75 kDa (containing AMPKα and phospho-AMPKα Thr^172^) and (3) 75 to 250 kDa (containing PGC-1α). Each part of the membrane was incubated in 20 mL of blocking buffer (TBST containing 5% w/v semi-skimmed milk powder) for 1h at room temperature. The membranes were then incubated with rabbit anti-PGC-1α (1:1,000) (AbClonal) or anti-AMPKα (1:1,000) (Cell Signaling Technology) in TBST containing 0.5% w/v semi-skimmed milk powder overnight or anti-GAPDH (1:10,000) (Cell Signaling Technology) for 1 h at 4°C under stirring. Membranes were washed 3 times for 10 min in TBST containing 0.5% w/v semi-skimmed milk powder and incubated with goat anti-rabbit IgG, horseradish peroxidase (HRP)-linked antibody (1:2,000 dilution) (Cell Signaling Technology) 1 h at 4°C. Finally, blots were washed 4 times for 10 min in TBST and rinsed twice for 10 min in TBS. They were exposed to enhanced chemiluminescence (ECL) reagents (Clarity^TM^ Western ECL Substrate, BioRad) according to the manufacturer’s protocol. The ECL signal was acquired from 20 s to 5 min. Proteins were quantified using a Vilber-Lourmat Fusion SL image acquisition system. Rabbit anti-AMPKα antibodies of the membrane (2) were stripped using a Re-blot Plus kit (Millipore) and then incubated in 20 mL of blocking buffer for 1 h at room temperature. The membrane was then incubated with rabbit anti-phospho-AMPKα Thr^172^ (1:1,000) (Cell Signaling Technology) in TBST containing 0.5% w/v semi-skimmed milk powder overnight at 4°C. The rest of the Western Blot protocol was the same as described above. Finally, the p-AMPKα/AMPKα ratio was calculated and PGC-1α protein quantification was normalized using GAPDH, considered as a reference protein.

### Enzymatic activities

All measurements were performed at 37°C and determined using a plate reader (SAFAS Xenius, Monaco). All samples were measured in duplicate. For each muscle (*soleus* and EDL), right and left muscles were ground together in liquid nitrogen to obtain a homogeneous tissue powder.

#### Aerobic metabolism enzyme activities

*Citrate synthase (CS) activity*. Samples of 25 mg of frozen muscle were homogenized in 1.5 mL Tris HCl buffer (0.1 M, pH 8.1, 4°C) with a Polytron. The homogenate was then collected and used immediately for analysis. Measurements of CS activity were realized on 6 μL of tissue extract and were made by an indirect method [31] using 5,5-dithio-bis-2-nitrobenzoic acid (DTNB). CS activity was measured at 412 nm and expressed in nmol DNTB reduced/min/mg wet tissue.

*Cytochrome c oxidase (COX) activity*. Samples of 70 mg of frozen muscle were homogenized with a Polytron homogenizer in 1 mL of extraction buffer (100 mM Tris, 2 mM EDTA and 2 mM DTE, pH 7.4, 4°C). The homogenate was centrifuged at 12,000 *g* for 20 min at 4°C. COX activity was determined on 50 μL of supernatant at 550 nm using 2 mM reduced cytochrome *c* and 330 mM sodium phosphate buffer [32]. COX activity was expressed in nmol cytochrome *c* oxidized/min/g wet tissue.

#### Anaerobic metabolism enzyme activity

*Lactate dehydrogenase (LDH) activity*. Samples of 70 mg of frozen muscle were homogenized with a Polytron in 1 mL of extraction buffer (100 mM Tris, 2 mM EDTA and 2 mM DTE, pH 7.4, 4°C). The homogenate was centrifuged at 12,000 *g* for 20 min at 4°C. LDH activity was determined on 2 μL of the resulting supernatant at 340 nm using 40 mM sodium pyruvate and 40 mM nicotinamide adenine dinucleotide (NADH) [33]. Activity was calculated based on oxidation of NADH and expressed in μmol oxidized NADH/min/g wet tissue.

#### Antioxidant enzyme activities

Samples of 70 mg of frozen muscles were homogenized with a Polytron homogenizer in 1 mL of extraction buffer (75 mM Tris and 5 mM EDTA, pH 7.4, 4°C). After a centrifugation at 12,000 *g* for 10 min at 4°C, superoxide dismutase (SOD), catalase (CAT) and glutathione peroxidase (GPx) activities were determined on the resulting supernatant.

**SOD activity** was assessed at 480 nm using an indirect method that inhibits the adrenaline to adenochrome reaction with the xanthine/hypoxanthine reaction as a superoxide anion producer [34] on 16 μL of supernatant. One unit (U) of SOD activity corresponds to the amount of sample needed to cause 50% inhibition relative to the control without tissue. SOD activity was expressed in U/g wet tissue.

**GPx activity** was measured at 340 nm with an indirect method adapted from Ross *et al*. (2001) by Farhat *et al*. (2015) [35, 36] using 25 μL or 100 μL of *soleus* or EDL supernatant, respectively. Briefly, activity was determined from the decrease of NADPH induced by a coupled reaction with glutathione reductase. GPx activity was expressed in μmol NADPH oxidized/min/g wet tissue.

**CAT activity** was determined at 240 nm through its capacity to transform hydrogen peroxide (H_2_O_2_) into water and oxygen [37]. The addition of 200 mM H_2_O_2_ to the 40 μL of tissular supernatant initiated the reaction. CAT activity was expressed in nmol H_2_O_2_/min/g wet tissue.

### Oxidative stress marker

Total plasmatic 8-isoprostane (free and esterified in lipids) was measured in duplicate using an Elisa kit (Cayman Chemical, Ann Arbor, Michigan, USA) according to the manufacturer’s protocol. During sampling, 0.005% of butylated hydroxytoluene (BHT) was added to all plasma collection tubes intended for this measurement to prevent oxidative formation of 8-isoprostane after collection.

All plasmatic samples were hydrolysed using 15% KOH and incubated for 60 min at 40°C and then neutralized with potassium phosphate buffer. A further step of purification was necessary with ethanol. Finally, samples were extracted using ethyl acetate containing 1% methanol and SPE Cartridges (C-18) (Cayman Chemical). After 18 h of incubation, 8-isoprostane plasmatic concentration was measured at 410 nm and expressed in pg/mL plasma.

### Statistics

All results are given as means ± standard error of the mean (SEM). All statistics were performed using Statistica v.12 software (StatSoft, Paris, France). Normality of distributions was tested using the Shapiro-Wilk test. Adapted tests were then performed (Kruskal-Wallis, one-way analyses of variance (ANOVA), two-way ANOVA or ANOVA for repeated measures). Kruskal-Wallis, ANOVA and two-way ANOVA were followed by Mann and Whitney, Tukey and Bonferroni post-hoc tests, respectively. The significance threshold was set at p < 0.05 and differences between groups indicated on the figures by different letters (a and b) or by symbols.

A principal component analysis (PCA) was performed on levels of all mRNAs using R software and the *FactoMineR* package.

## Results

### Maximal aerobic speed (MAS)

**Fig 1:**
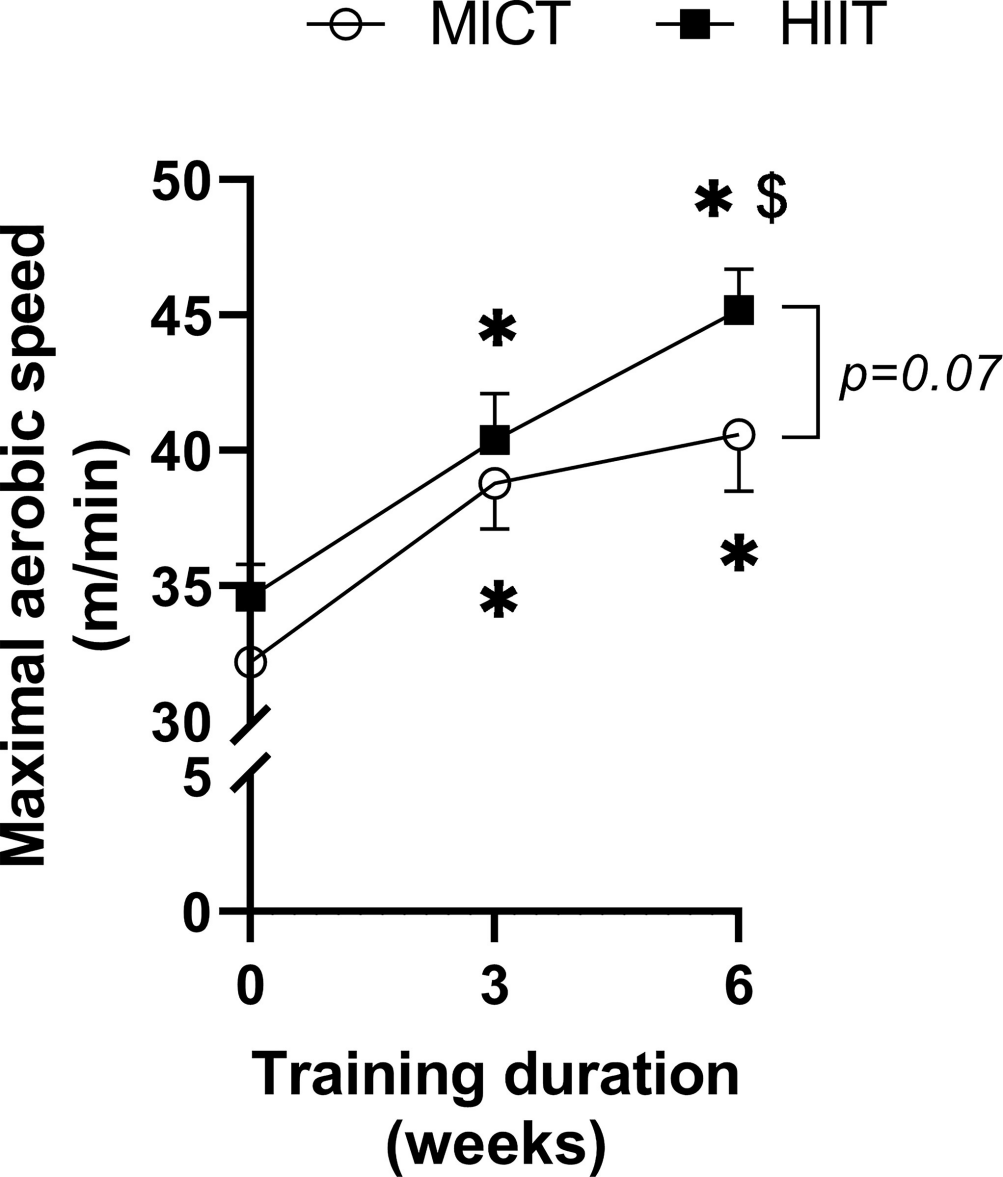
Effect of MICT and HIIT on maximal aerobic speed (MAS) as a function of training duration. Values of MAS are means ± SEM. In a same experimental group (MICT or HIIT), * indicates a significant difference from the MAS before starting the training (p < 0.05) and **$** indicates a significant difference from the MAS after three weeks of training (p < 0.05). No significant differences were observed between MICT and HIIT.

To normalize individual training and to evaluate training efficiency, the MAS of trained animals (MICT and HIIT) was measured before training and after three and six weeks of training (**Fig 1**). Two-way ANOVA revealed a significant time effect (p < 0.001), but no training effect (p = 0.07) and no interaction between time and training (p = 0.59).

Before starting the treadmill training, both MICT and HIIT groups had similar MAS values: 32.3 ± 0.03 m/min and 34.6 ± 1.2 m/min, respectively. For the MICT group, MAS was only significantly increased after three weeks of training (38.8 ± 1.7 m/min; p = 0.009) and stabilized after six weeks of training (40.6 ± 2.1 m/min). MAS rose continuously for the HIIT group after three (40.4 ± 1.7 m/min; p = 0.009) and six weeks of training (45.2 ± 1.5 m/min; p = 0.049). The last MAS values measured tended to be higher after HIIT than after MICT (p = 0.07).

### Rat monitoring, morphometric and systemic measurements

**Table 2** summarizes the monitoring during the experiment, morphometric and systemic measurements of the experimental groups. After six weeks of training, the training volume of HIIT was 2-fold lower than that of MICT with an approximately 1.6-fold lower cumulative running distance and time. No training effects were shown on the weight gain and total calorie intake of rats during the six weeks of training. MICT induced a decrease of the adipose index compared with UNTR and HIIT regimes. Otherwise, no significant effects of training were observed for the other measurements (body weight, naso-anal body length, BMI, circumference index and Lee index). Neither MICT nor HIIT modified heart rate, mean, systolic and diastolic arterial blood pressures, hematocrit or cutaneous vascular conductance of the rats.

**Table 2:** Monitoring, morphometric and systemic parameters of UNTR, MICT and HIIT experimental groups.

			UNTR *n* = 7	MICT *n* = 8	HIIT *n* = 8
**Monitoring during experimental period**	Total running distance (km)		-	36.89 ± 0.83 **a**	21.59 ± 0.37 **b**
	Total running time (min)		-	1715.99 ± 17.10 **a**	1087.56 ± 10.62 **b**
	Total training volume (A.U.)		-	1875.44 ± 5.21 **a**	904.25 ± 8.98 **b**
	Weight gain (g)		116.29 ± 9.09	95.75 ± 5.65	104.88 ± 7.49
	Total calorie intake (kcal/day/100 g of rat body weight)		23.00 ± 0.75	22.84 ± 0.89	22.46 ± 0.64
**Morphometric parameters**	Body weight (g)		417.14 ± 14.62	372.63 ± 12.20	387.63 ± 12.26
	Naso-anal body length (cm)		24.93 ± 0.46	24.63 ± 0.40	24.88 ± 0.40
	Body mass index (g/cm^2^)		0.67 ± 0.02	0.61 ± 0.02	0.63 ± 0.02
	Circumference index		1.07 ± 0.01	1.03 ± 0.02	1.04 ± 0.01
	Lee index		2.99 ± 0.03	2.92 ± 0.04	2.93 ± 0.04
	Adiposity index		4.11 ± 0.42 **a**	2.33 ± 0.35 **b**	3.61 ± 0.28 **a**
**Systemic parameters**	Heart rate (bpm)		373.39 ± 12.46	361.32 ± 9.62	377.68 ± 9.21
	Mean arterial blood pressure (mmHg)		122.88 ± 2.02	114.00 ± 4.78	119.57 ± 4.31
	Systolic arterial blood pressure (mmHg)		154.83 ± 2.44	145.31 ± 4.92	154.32 ± 4.34
	Diastolic arterial blood pressure (mmHg)		107.79 ± 1.78	99.26 ± 5.01	103.57 ± 4.14
	Hematocrit (% red blood cells)		47.96 ± 2.27	48.93 ± 3.57	48.24 ± 1.18
	LDF (% variation of CVC)	ACh	61.29 ± 2.93	55.02 ± 4.90	59.20 ± 5.69
		SNP	41.67 ± 6.61	45.35 ± 6.44	55.27 ± 4.85

Total training volume was calculated as the product of exercise intensity (% of MAS) and total training duration over the six weeks. Weight gain and total calorie intake of each rat were measured during the six weeks of the experiment. Morphometric and systemic parameters were measured at the end of the experiment. Values are means ± SEM. Different letters indicate significant differences between groups (p<0.05). UNTR: untrained; MICT: moderate-intensity continuous training; HIIT: high-intensity interval training; LDF: laser doppler flowmetry; CVC: cutaneous vascular conductance.

### Proportions of myosin heavy chain isoform mRNAs in the untrained group

To verify the proportions of myosin heavy chain (MHC) isoform in *soleus* and EDL muscles, **Table 3** gives their mRNA percentages determined in the UNTR group. *Soleus* samples were mainly composed of slow-twitch fibre type I MHC mRNA (86.5 ± 5.0%) and less than 2% of fast-twitch fibre types (IIx and IIb), whereas EDL ones principally consisted of fast-twitch fibre type IIx (46.6 ± 1.3%) and type IIb (47.2 ± 1.7%) MHC mRNAs.

**Table 3:** Distribution of myosin heavy chain isoform mRNAs in *soleus* and EDL muscles in the UNTR group.

	Myosin heavy chain isoform mRNAs proportion (%)			
Muscle (*n* = 6)	I	IIa	IIx	IIb
*Soleus*	86.5 ± 5.0	11.8 ± 5.0	0.5 ± 0.1	1.3 ± 0.2
**EDL**	0.4 ± 0.2	5.7 ± 0.8	46.6 ± 1.3	47.2 ± 1.7

Values are means ± SEM.

### mRNA correlations in *soleus* and EDL muscles

**Fig 2:**
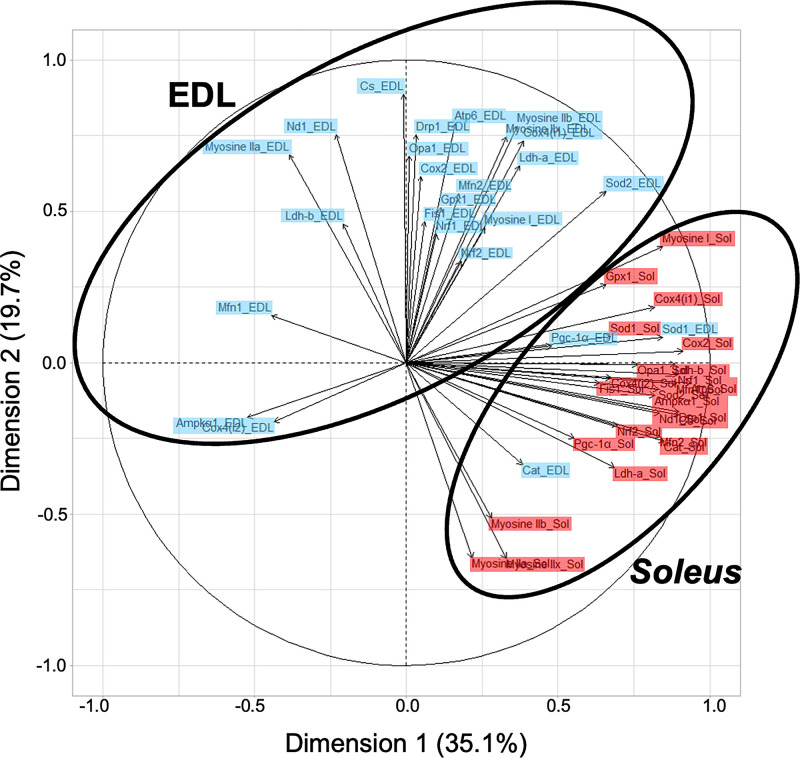
Principal component analysis (PCA) performed using all mRNA levels studied in *soleus* (in red, *n* = 21) and EDL (in blue, *n* = 21) muscles of the MICT and HIIT groups. See **Table 1** for more details.

Based on the mRNA data from the two training protocols, the principal component analysis (PCA) indicated two distinct clusters that correspond globally to *soleus* and EDL muscles (**Fig 2**). The first principal component accounted for 35.1% of the transcriptomic variability among the genes and the second principal component accounted for 19.7%.

### Influence of MICT and HIIT on skeletal muscle transcripts

#### AMPK–PGC-1α signalling pathway

**Fig 3:**
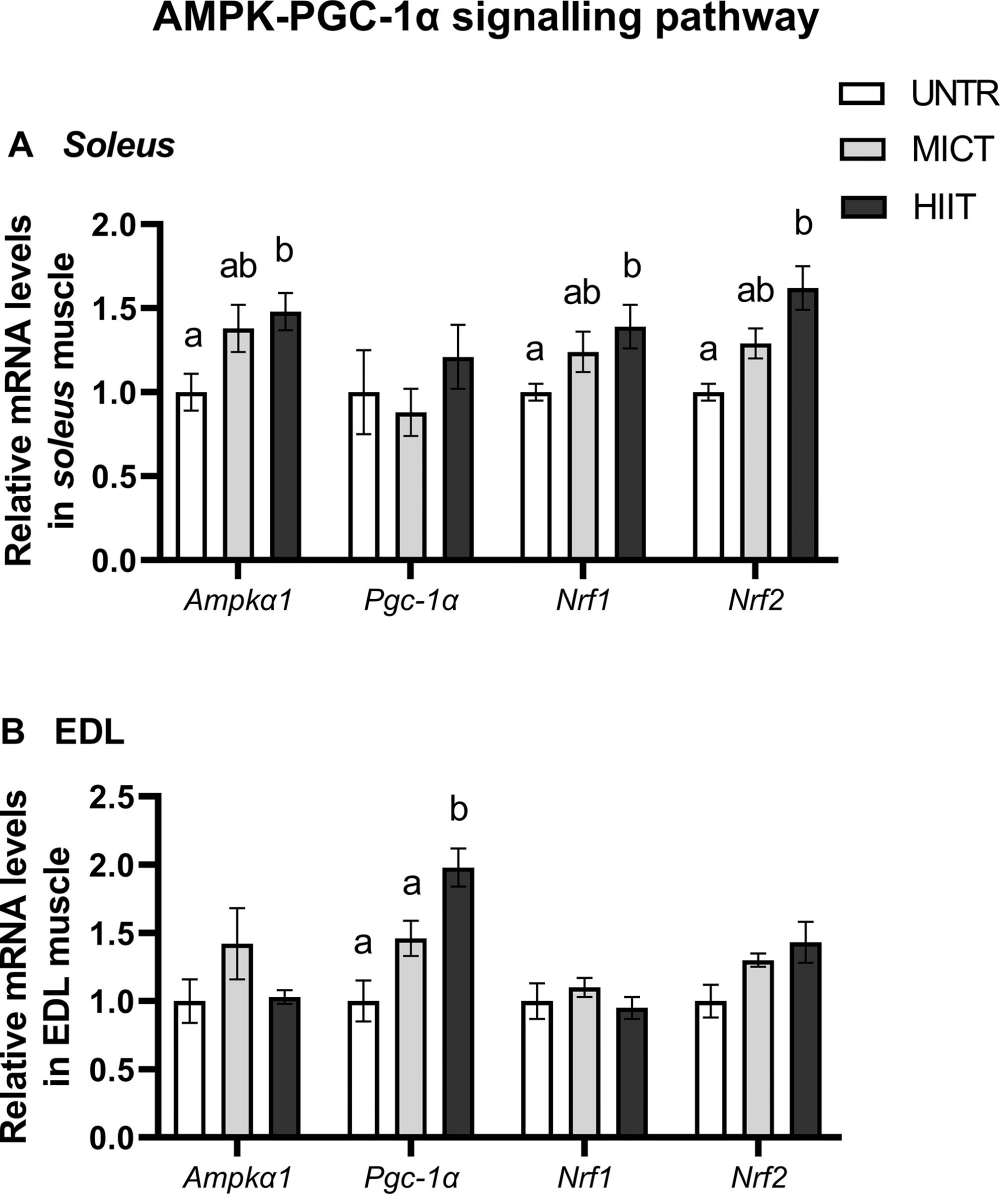
Effects of MICT and HIIT on *Ampkα1*, *Pgc-1α*, *Nrf1* and *Nrf2* mRNA levels in *soleus* (A) and EDL (B) muscles. UNTR: *n* = 6; MICT: *n* = 7; HIIT: *n* = 8. Results are expressed as fold change compared with UNTR, which is set at 1. Results are means ± SEM. Bars with different letters indicate groups that are significantly different (p < 0.05).

In *soleus*, HIIT increased *Ampkα1*, *Nrf1* and *Nrf2* mRNA levels compared with UNTR, but no effects of training protocol were observed on *Pgc-1α* mRNA (**Fig 3A**). In EDL, in contrast, only *Pgc-1α* mRNA content was significantly up-regulated by HIIT compared with UNTR (p = 0.0007) and MICT (p = 0.045) (**Fig 3B**).

#### Mitochondrial functioning

**Fig 4:**
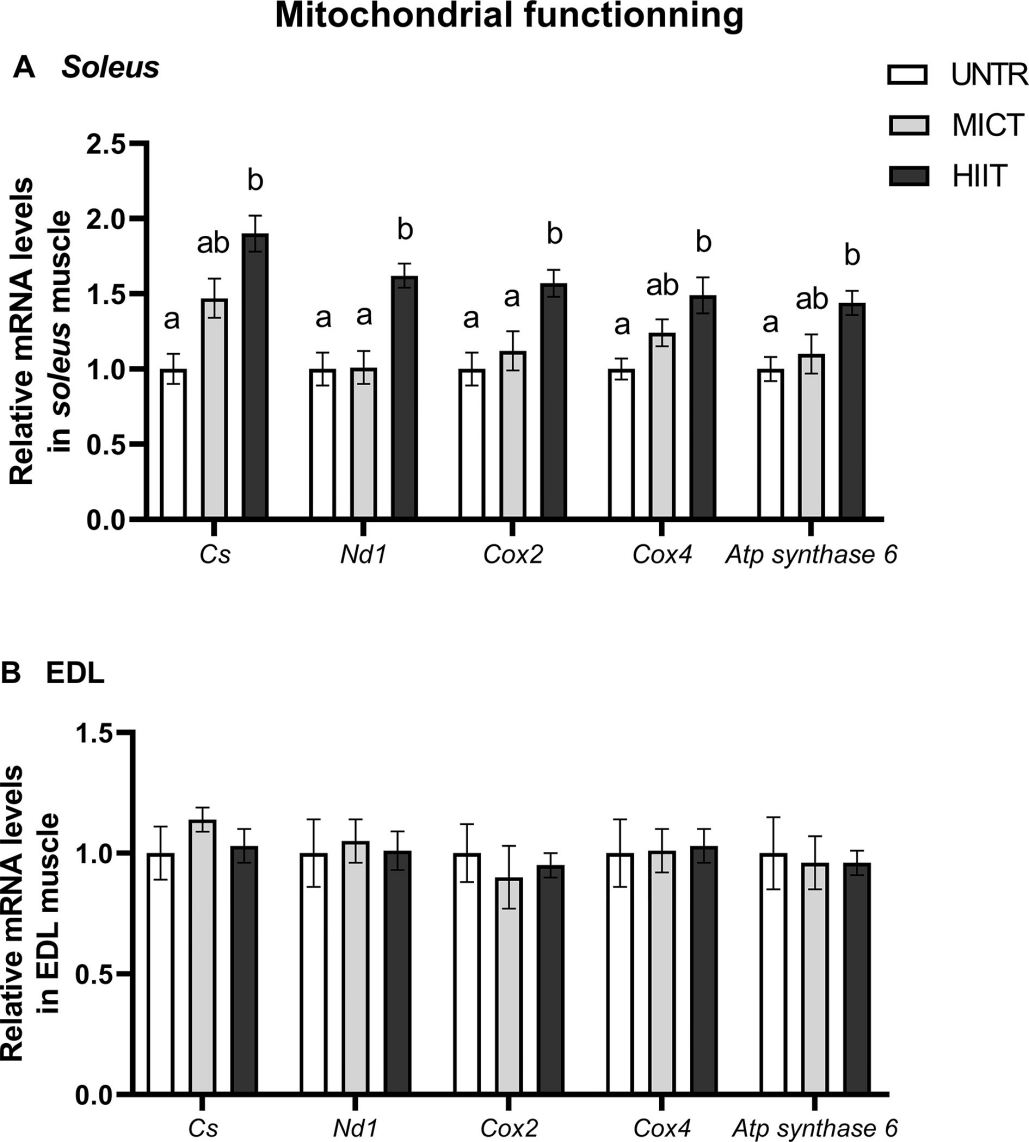
Effects of MICT and HIIT on *citrate synthase* (*Cs*), *NADH dehydrogenase 1* (*Nd1*), *cytochrome c oxidase (Cox) 2* and *4* and *Atp synthase 6* mRNA levels in *soleus* (A) and EDL (B) muscles. UNTR: *n* = 6; MICT: *n* = 7; HIIT: *n* = 8. Results are expressed as fold change compared with UNTR, which is set at 1. Results are means ± SEM. Bars with different letters indicate groups that are significantly different (p < 0.05).

In *soleus*, the *Cs* mRNA content was significantly increased by almost 2-fold (p = 0.0005) by HIIT. HIIT also increased *Nd1* and *Cox2* mRNA contents compared with UNTR and MICT, and transcription of *Cox4* and *Atp synthase 6* were stimulated by HIIT compared with UNTR (**Fig 4A**). Neither MICT nor HIIT had significant effects on the mRNA levels related to mitochondrial functioning in EDL (**Fig 4B**).

#### Mitochondrial dynamics

**Fig 5:**
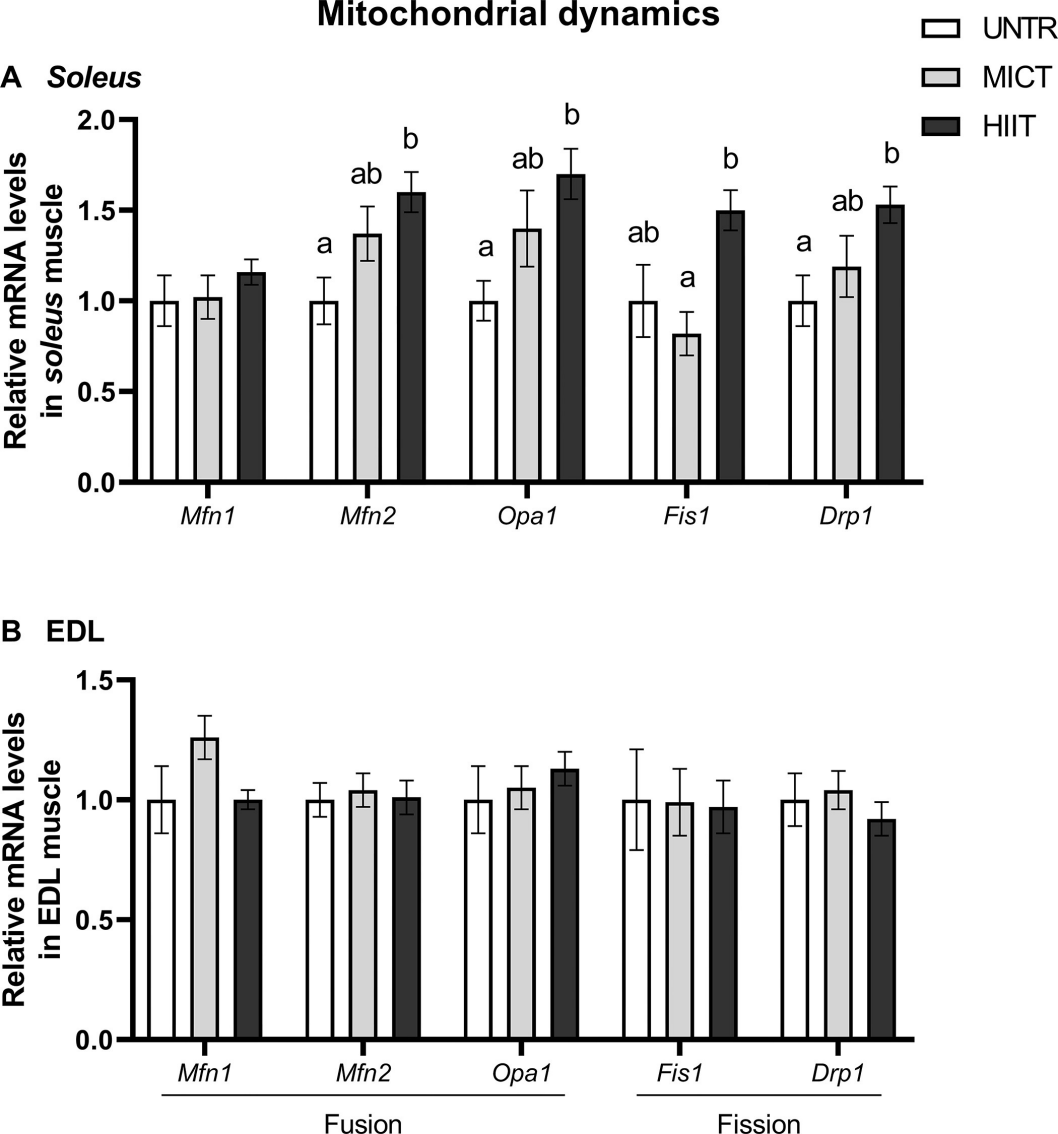
Effects of MICT and HIIT on mRNA levels related to mitochondrial fusion: *mitofusin* (*Mfn*) *1* and *2* and *optic atrophy protein* (*Opa1*); and fission: *fission protein 1* (*Fis1*) and *dynamin-related protein 1* (*Drp1*) in *soleus* (A) and EDL (B) muscles. UNTR: *n* = 6; MICT: *n* = 7; HIIT: *n* = 8. Results are expressed as fold change compared with UNTR, which is set at 1. Results are means ± SEM. Bars with different letters indicate groups that are significantly different (p < 0.05).

In *soleus*, *Mfn2*, *Opa1* and *Drp1* mRNA levels in the HIIT group were at least 50% higher (p < 0.05) than in the UNTR group. HIIT up-regulated *Fis1* transcription compared with MICT (**Fig 5A**). No effects of training protocol were observed on these mitochondrial dynamics genes in EDL (**Fig 5B**).

#### Antioxidant defences

**Fig 6:**
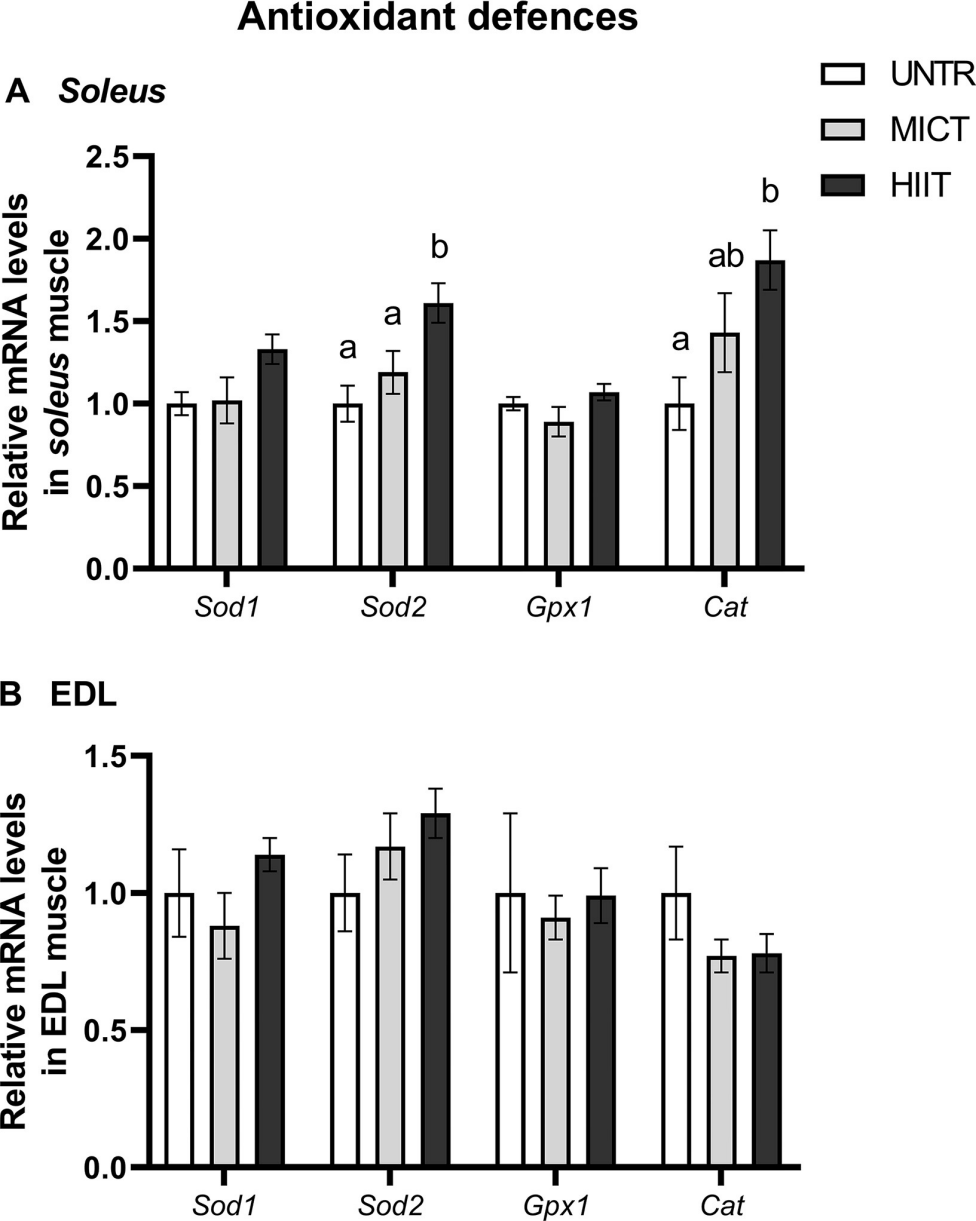
Effects of MICT and HIIT on mRNA levels related to antioxidant defences, *superoxide dismutase* (*Sod*) *1* and *2*, *glutathione peroxidase 1* (*Gpx1*) and *catalase* (*Cat*), in *soleus* (A) and EDL (B) muscles. UNTR: *n* = 6; MICT: *n* = 7; HIIT: *n* = 8. Results are expressed as fold change compared with UNTR, which is set at 1. Results are means ± SEM. Bars with different letters indicate groups that are significantly different (p < 0.05).

In *soleus*, *Sod2* mRNA level was higher in the HIIT group than in the UNTR (p = 0.004) and MICT (p = 0.04) groups. HIIT increased the *Cat* mRNA content compared with UNTR (p = 0.02) (**Fig 6A**). In EDL, training had no effects on antioxidant defence mRNAs (**Fig 6B**).

#### Myosin heavy chain

**Fig 7:**
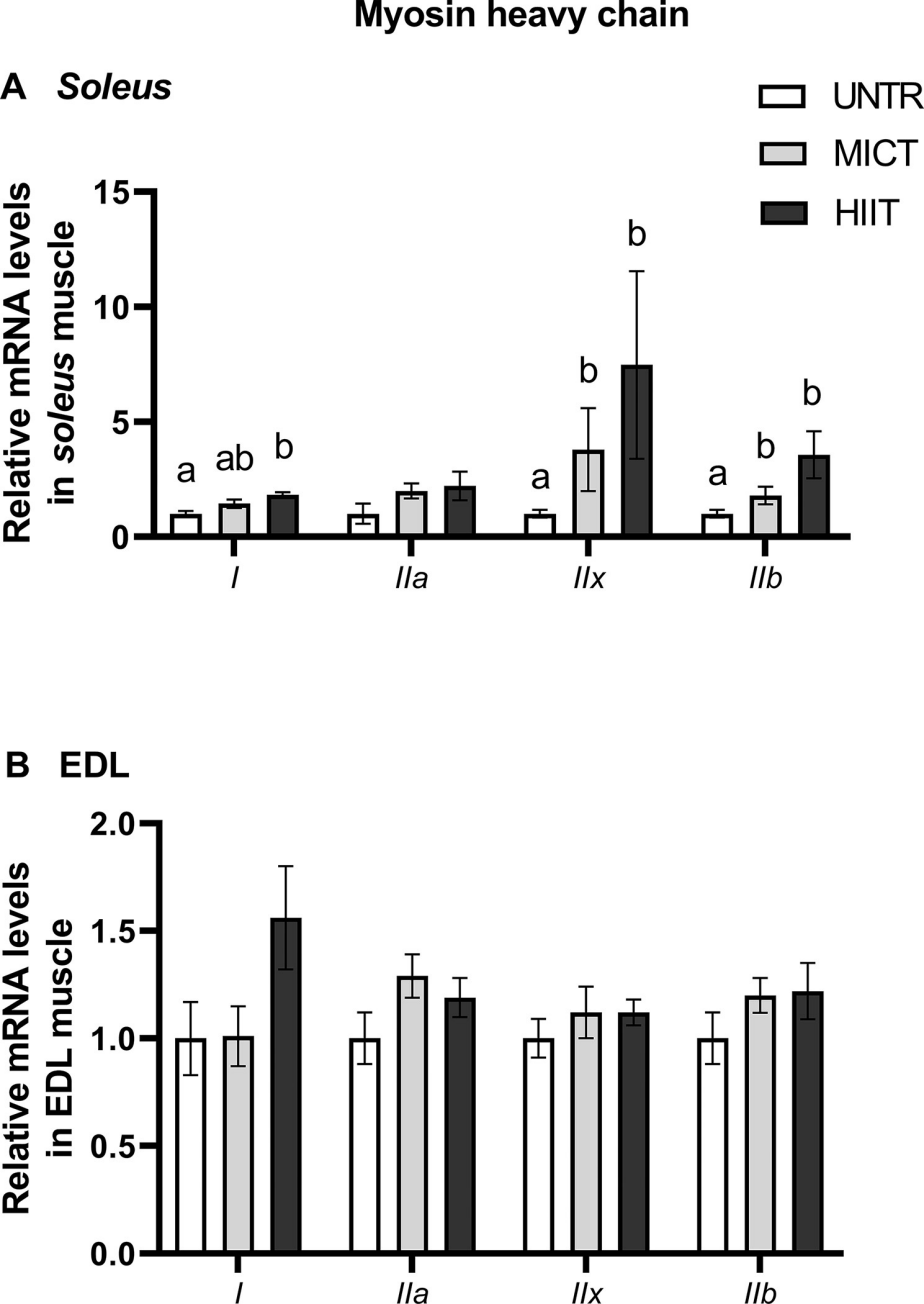
Effects of MICT and HIIT on *myosin heavy chain I*, *IIa*, *IIx* and *IIb* mRNA levels in *soleus* (A) and EDL (B) muscles. UNTR: *n* = 6; MICT: *n* = 7; HIIT: *n* = 8. Results are expressed as fold change compared with UNTR, which is set at 1. Results are means ± SEM. Bars with different letters indicate groups that are significantly different (p < 0.05).

In *soleus*, HIIT increased the transcription of types I, IIx and IIb myosin heavy chain (MHC) mRNAs compared with UNTR. MICT stimulated the mRNA levels of MHC types IIx and IIb compared with UNTR (**Fig 7A**). Neither MICT nor HIIT induced significant changes in MHC I, IIa, IIx or IIb mRNA compositions in EDL (**Fig 7B**).

#### Lactate dehydrogenase subunits

**Fig 8:**
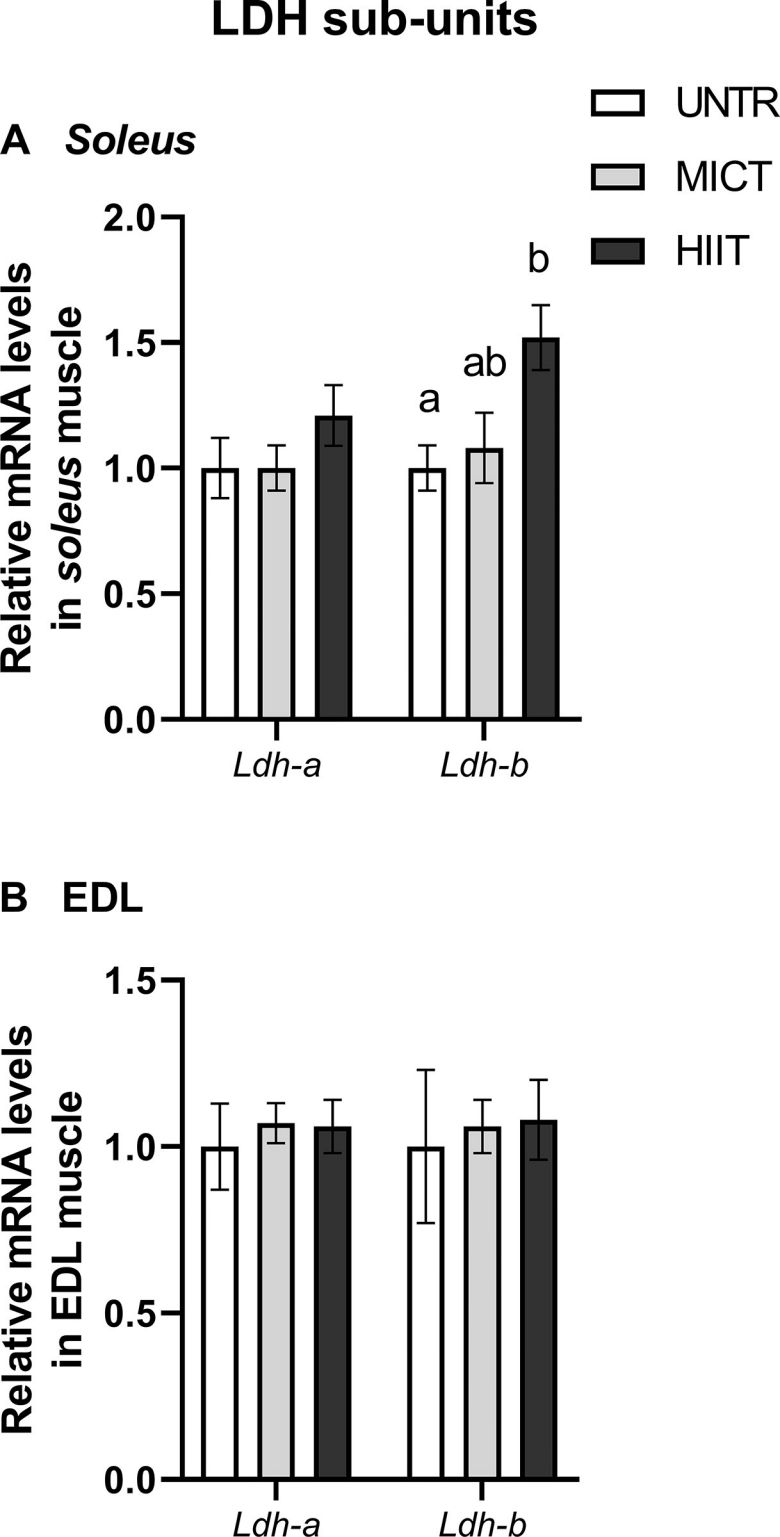
Effects of MICT and HIIT on *lactate dehydrogenase* (*Ldh)-a* and *-b* subunit mRNA levels in *soleus* (A) and EDL (B) muscles. UNTR: *n* = 6; MICT: *n* = 7; HIIT: *n* = 8. Results are expressed as fold changes compared with UNTR, which is set at 1. Results are means ± SEM. Bars with different letters indicate groups that are significantly different (p < 0.05).

In *soleus*, HIIT stimulated *Ldh-b* transcription compared with UNTR (p = 0.01) but had no effect on *Ldh-a* mRNA content (**Fig 8A**). In EDL, neither type of training modified either of these two gene contents (**Fig 8B**).

### Western blot: PGC-1α and p-AMPKα/AMPKα protein contents

**Fig 9:**
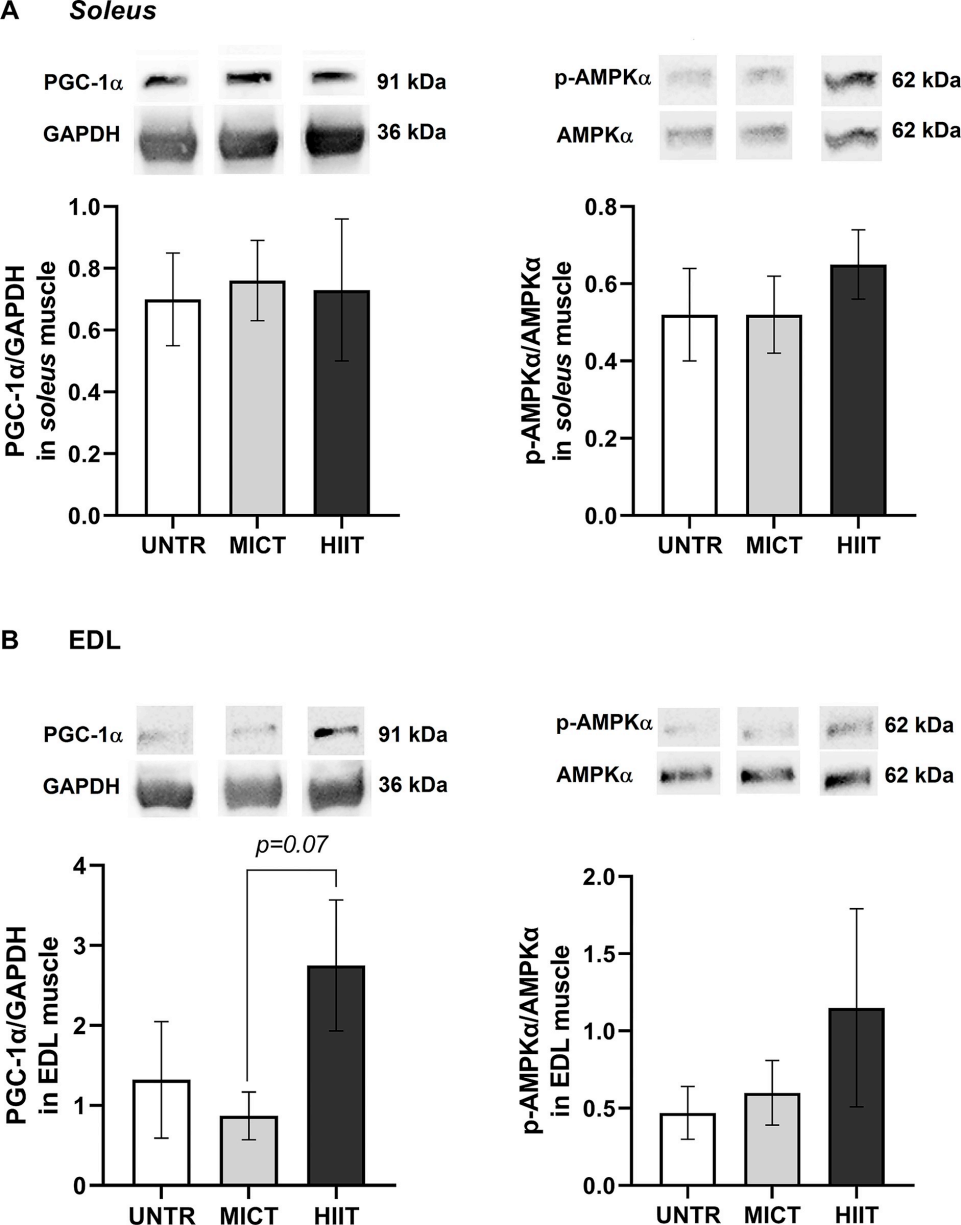
Effects of MICT and HIIT on PGC-1α and p-AMPKα/AMPKα ratio protein contents in *soleus* (A) and EDL (B) muscles. PGC-1α was normalized with GAPDH. *Soleus*: UNTR: *n* = 7; MICT: *n* = 6 and HIIT: *n* = 8. EDL: UNTR: *n* = 6; MICT: *n* = 7 and HIIT: *n* = 8. Results are means ± SEM. No significant differences were observed between groups.

Neither MICT nor HIIT had significant effects on PGC-1α or p-AMPKα/AMPKα ratio protein expressions in *soleus* or EDL (**Fig 9**).

### Enzymatic activities

In *soleus*, training produced no significant effects on the enzymatic activities of CS, COX, LDH, SOD, GPx or CAT (**Table 4**).

**Table 4:** Effects of MICT and HIIT on CS, COX, LDH, SOD, GPx and CAT activities in *soleus* and EDL muscles.

	*Soleus*			EDL		
	UNTR *n* = 7	MICT *n* = 8	HIIT *n* = 8	UNTR *n* = 7	MICT *n* = 8	HIIT *n* = 8
**CS (nmol DTNB reduced/min/mg WT)**	7.1 ± 0.8	10.1 ± 1.2	8.0 ± 0.7	8.1 ± 0.3	8.6 ± 0.2	7.6 ± 0.3
COX (nmol cyt. *c* oxidized/min/g WT)	28.5 ± 3.3	35.2 ± 4.2	35.5 ± 4.7	20.7 ± 1.2	15.7 ± 1.5	19.7 ± 3.2
**LDH (μmol oxidized NADH/min/g WT)**	148.0 ± 6.2	143.9 ± 9.1	157.4 ± 8.5	704.4 ± 88.6	677.9 ± 32.2	694.8 ± 23.4
**SOD (U/g WT)**	491.6 ± 54.0	513.4 ± 57.4	586.7 ± 50.4	289.0 ± 52.5	356.1 ± 68.8	361.4 ± 58.9
**GPx (μmol NADPH oxidized/min/g WT)**	7.9 ± 0.3	7.4 ± 0.2	7.8 ± 0.4	0.9 ± 0.1 **a**	1.0 ± 0.04 **a**	1.2 ± 0.1 **b**
CAT (nmol H_2_O_2_/min/g WT)	1151.1 ± 97.2	1019.9 ± 80.7	944.8 ± 77.0	216.1 ± 22.5	236.9 ± 24.6	209.5 ± 10.2

Results are means ± SEM. Different letters indicate significant differences between groups (p < 0.05).

UNTR: untrained; MICT: moderate-intensity continuous training; HIIT: high-intensity interval training; CS: citrate synthase; COX: cytochrome *c* oxidase; LDH: lactate dehydrogenase; SOD: superoxide dismutase; GPx: glutathione peroxidase; CAT: catalase; WT: tissue weight.

In EDL, GPx activity was ~26% higher after HIIT compared with UNTR (p = 0.006) (**Table 4**).

### Oxidative stress marker

Neither MICT nor HIIT modified plasmatic 8-isoprostane concentration (**Table 5**), considered as an oxidative stress marker.

**Table 5:** Effects of MICT and HIIT on plasmatic 8-isoprostane concentrations.

	UNTR *n* = 7	MICT *n* = 8	HIIT *n* = 8
**8-isoprostane (pg/mL)**	140.1 ± 32.3	96.4 ± 18.9	110.9 ± 21.5

Results are means ± SEM. No significant differences were observed between groups.

UNTR: untrained; MICT: moderate-intensity continuous training; HIIT: high-intensity interval training.

## Discussion

The main aim of the present study was to compare the effects of the MICT and HIIT protocols on aerobic (AMPK–PGC-1α signalling pathway, mitochondrial biogenesis, antioxidant defences) and anaerobic (lactate dehydrogenase) metabolic processes in two muscles with different typologies: *soleus* and *extensor digitorum longus* (EDL). To make the approach integrative, training effects were also examined at the whole organism level through measurements of maximal aerobic speed (MAS), morphometric and systemic parameters.

The three main findings were: 1) endurance performance (MAS) and oxidative capacities (muscle transcripts and proteins) were more greatly stimulated by HIIT than by MICT; 2) transcription was generally activated more in *soleus* muscle than in EDL in response to both MICT and HIIT; 3) solely on the basis of the mRNA results determined after training, two distinct mRNA profiles related to the muscle typology (*soleus* and EDL) were revealed.

One of the present challenges in human health is to define a training protocol that confers overall health benefits through, in part, the stimulation of oxidative and antioxidant capacities in skeletal muscle and which fits into today’s lifestyle. In human skeletal muscle, studies showed that HIIT would be more efficient than MICT for stimulating mitochondrial biogenesis [8] and functioning [38]. Exercise intensities between 50 and 70% of maximal oxygen uptake ($\dot{V}O_{2}\max$) for MICT and between 85 and 95% $\dot{V}O_{2}\max$ for HIIT are commonly applied to improve aerobic capacity in human [39]. In the present study, the exercise intensities applied are in accordance with those commonly used. After 3 weeks of training, the MAS was significantly improved with both types of exercise used, showing the efficiency of our protocols. Then, during the following three weeks of training, while the MAS remained stabilized with MICT, it tended to continue to increase with HIIT (p = 0.07, MICT *vs* HIIT). Our results are consistent with two recent studies using work-matched training MICT and HIIT in healthy rats [9, 40]. Because the MAS is related to $\dot{V}O_{2}\max$, we can suggest that HIIT is efficient for improving $\dot{V}O_{2}\max$. However, we cannot exclude an improvement in MAS also related to increased anaerobic capacities because HIIT is characterized by repeated bouts at high intensity (85 to 90% of MAS) [9, 40]. In human, a greater gain in $\dot{V}O_{2}\max$ has also been reported with HIIT compared with MICT work-matched training programs [41]. So, in the present study, despite a training volume (product of exercise intensity and total training duration) exactly 2-fold lower for HIIT compared with MICT, the rats’ physical performance was improved. This suggests the importance of the intensity level and/or of the periods of recovery between the bouts of exercise [42].

Among the parameters examined, the adiposity index, an indicator of obesity-related disorders [43], decreased significantly with MICT but not with HIIT. It is known that prolonged moderate intensity exercise or a high training volume (as with MICT) enhance fatty acid utilization and particularly when exercise intensity is higher than 50–65% $\dot{V}O_{2}\max$, fatty acid oxidation shifts to glucose oxidation [44]. Thus, we supposed that six weeks of MICT increased lipolysis more than HIIT did by mobilizing fatty acids for ATP production. Otherwise, no effect of training was observed on cardiovascular and systemic parameters such as heart rate, blood pressures, microvascular endothelial function in peripheral circulation and hematocrit. Recent studies demonstrated that four or six weeks of MICT or HIIT had no effects on healthy Wistar rats’ systolic and diastolic blood pressures or on heart rate [45, 46]. Few studies have explored the cutaneous microvascular endothelial function after HIIT training in murine models. In human, Lanting *et al*. (2017) suggested that exercise training could improve this in people with microvascular disease and healthy physically inactive adults, but not in healthy adults who are physically active [47]. Therefore, the absence of change in the cutaneous microcirculation of the healthy and active rats in our study seems relevant.

At the skeletal muscle level, the AMPK–PGC-1α signalling pathway is recognized as one of the most potent stimulators of mitochondrial biogenesis in skeletal muscle. PGC-1α is involved in many processes by stimulating the two nuclear respiratory factors (NRF) 1 and 2, which are transcription factors involved in the regulation of mitochondrial biogenesis and antioxidant systems, respectively [48]. PGC-1α is also known to be a regulator of mitochondrial dynamics including fusion and fission processes. Finally, skeletal muscle fibre type determination is also under the influence of PGC-1α function [49].

Two muscles were explored for their distinct fibre type composition: the slow-twitch *soleus*, composed of 80% of oxidative type I fibres and the fast-twitch EDL, composed exclusively of type II fibres using mainly anaerobic metabolism [50]. These two muscles also differ in mitochondrial content because type I fibres contain a much larger volume of mitochondria than type II fibres [25]. In the present study, the muscle metabolic specificities are related by the effects of training protocols on levels of transcripts involved in metabolic processes (AMPK–PGC-1α signalling pathway and antioxidant systems). As a whole, the mRNA responses clearly differentiate *soleus* and EDL, as shown by the two clusters of the PCA. It is important to remember that these responses are related to training effects and not to acute exercise effects because the post-training muscle samples were taken at least 48 hours after the last training session.

One of the important findings of our study is that *soleus* muscle had far more numerous responses to training than EDL. Because *soleus* muscle is mainly oxidative, we can suggest that both the MICT and HIIT training protocols used here required more slow-twitch fibres than fast-twitch fibres. Kryściak *et al*. (2018) also showed that four and eight weeks of endurance training stimulated more transcripts and proteins related to mitochondrial biogenesis in slow *gastrocnemius* fibres than in fast *gastrocnemius* fibres in rats [51].

In *soleus*, HIIT upregulated most of the studied mRNAs involved in the AMPK–PGC-1α signalling pathway, mitochondrial functioning and dynamics, and antioxidant defences compared with MICT.

Surprisingly, despite the numerous modified transcripts in *soleus*, the PGC-1α transcript was unchanged after training exercise. Some authors showed that *Pgc-1α* mRNA levels increase during the first exercise session and decrease with each subsequent session despite maintaining exercise intensity [52]. Western blot results showed that neither PGC-1α protein content nor p-AMPKα/AMPKα ratio changed significantly with training, while the AMPK transcript was increased with HIIT. Miller *et al*. (2016) has shown that exercise-induced mRNAs do not necessarily translate into proteomic changes [53]. Moreover, post-transcriptional or -translational regulatory processes may have already occurred, as mentioned by Robinson *et al*. (2017) [54]. One of these processes, or a combination of them, could explain the activation of the transcripts involved in mitochondrial functioning and antioxidant responses without there being a change in proteins.

In *soleus*, MICT and HIIT differentially stimulate genes involved in mitochondrial fusion (*Mfn1* and *2* and *Opa1*), fission (*Fis1* and *Drp1*) and functioning (*Cs* and OXPHOS subunit complexes encoded by nuclear, *Cox4*, or mitochondrial, *Nd1*, *Cox2*, *Atp synthase 6*, genomes). Transcript responses in terms of mitochondrial dynamics conform with those of Perry *et al*. (2010), who showed that two weeks of high-intensity training increased MFN1, Fis-1, DRP-1 and COX4 protein expressions, and *Cs* and *Cox4* transcript levels in the human *vastus lateralis* muscle [52]. Taken together, these observations suggest that high-intensity training regulates mitochondrial quantity and quality so as to increase oxidative capacity in skeletal muscle. However, no changes were observed in CS or COX enzymatic activities. In the same way as for PGC-1α, it is possible that regulatory have processed after mRNAs production that could explain a training effect on mRNA levels but not on enzymatic activities [54, 55]. In the literature, a concomitant increase of *Cs* mRNA and its activity was shown in rat *gastrocnemius* after only 5 and 10 days of moderate training [38]. The difference in CS adaptations between the studies could also be explained by differences in training duration, time before sampling and, particularly, muscle type. The *gastrocnemius* muscle is often studied because it is greatly involved in treadmill running. Its composition of mixed fibres certainly facilitates its metabolic adaptations, which may be greater than in *soleus* (mainly composed of type I fibres).

During physical exercise, mitochondria are among the main sources of reactive oxygen species (ROS; [56]). These molecules are necessary for cellular process regulation but are harmful at high levels. ROS levels, therefore, need to be regulated by antioxidant mechanisms [57]. Enzymatic antioxidant defence mRNAs (*Cat* and mitochondrial *Sod2*) were increased by HIIT in *soleus* but without significant changes in antioxidant enzyme activities (SOD, GPx and CAT). Moreover, no oxidative stress occurred suggesting that the training protocols had no deleterious effects.

HIIT also induced a rise in myosin heavy chain (MHC) aerobic type I and anaerobic type IIx and IIb mRNA levels in *soleus*. The transcription stimulation of MHC isoforms type IIx and IIb can be surprising but it could be related to the relatively higher plasticity of *soleus* compared with EDL. At the muscle level, these modifications represent few changes because these two MHC isoform mRNAs represented less than 2% of the *soleus* total MHC isoforms. However, we should be cautious about these interpretations only based on mRNAs.

Concerning lactate dehydrogenase isoenzymes, LDH is a tetrameric enzyme composed of the subunits M and/or H encoded by *Ldh-a* and *Ldh-b* genes, respectively. LDH4M reduces pyruvate to lactate in tissues dependent on anaerobic glycolysis, whereas LDH4H permits lactate oxidation in tissues dependent on aerobic metabolism [58]. HIIT increased *Ldh-b* mRNA content with no changes in LDH activity (conversion of pyruvate to lactate) in *soleus*, suggesting higher aerobic capacities. In human, three weeks of training (70–85% $\dot{V}O_{2}\max$) induced an increase of *Ldh-b* mRNA levels and tended to decrease *Ldh-a* transcription in the *vastus lateralis* muscle [59]. It is important to remember that the PCA analysis, particularly in *soleus*, also highlights important correlations between the actors involved in AMPK–PGC-1α signalling pathway, mitochondria functioning and dynamics, antioxidant defences and LDH subunit mRNAs.

Because of the anaerobic phenotype of the EDL muscle, we could suppose that the anaerobic pathway might be activated during HIIT sessions. A higher LDH activity in EDL was observed compared with *soleus*. However, neither muscle showed an effect of training protocol on LDH enzymatic activity. Kristensen *et al*. (2015) also showed a greater increase in glycogen utilisation (preferential substrate for anaerobic glycolysis) in fast-twitch fibres after HIIT than in slow-twitch fibres whereas MICT induced no fibre type dependent difference [23].

In EDL muscle, contrary to *soleus*, *Pgc-1α* mRNA level was stimulated by HIIT and PGC-1α protein content tended to increase (p = 0.07) compared with MICT, suggesting an activated aerobic process in this muscle after six weeks of training. Otherwise, HIIT improved antioxidant defences in EDL, as shown by GPx activity stimulation. Although the studied transcripts were largely increased with HIIT in *soleus*, the sole change in protein content (PGC-1α content and GPx activity) was not observed in *soleus* but in EDL. This suggests that these two muscles could have different transcriptomic and/or post-transcriptomic and/or proteomic response kinetics during training exercise.

Few significant responses with HIIT were however observed in EDL compared with *soleus* (only Pgc*-1α* mRNA and GPx activity). EDL is mostly composed of type IIx and IIb MHC (more than 90%) and these fibres types would not contribute substantially to effort until an intensity of 100% $\dot{V}O_{2}\max$ was reached [7]. We can, therefore, suppose that 85–90% MAS might not be enough to totally recruit these fast-twitch fibre types.

One limitation of our study is the low number of protein measurements (enzymatic activity or Western Blot) after the training exercise. Indeed, to have a complete functional approach at the muscle level, it would have been interesting to make more protein quantifications and activity measurements to complement the transcript level results. In addition to transcriptional analysis, we chose to focus on some key elements involved in muscle adaptation to training (PGC-1α, AMPKα protein contents and antioxidant enzymatic activities) but were unfortunately limited to a restricted number of analyses by the quantity of tissue available. Another limitation is related to the choice of the training protocol parameters. The two training volumes were intentionally unmatched, as in the protocol of Brown *et al*. (2017) [60]. The HIIT training volume was deliberately chosen to be 2-fold lower than the MICT training volume, with running distance and time approximatively 1.6-fold lower. Such a protocol more accurately reflects how HIIT is performed in clinical practice, which has the main objective of maintaining or improving beneficial effects with a significantly reduced session duration. Nevertheless, it would be interesting in a future study to match volumes between training protocols to confirm that training intensity is a more important parameter than training volume for increasing mitochondrial oxidative capacity, a question that remains highly debated [61, 62]. Finally, for untrained rats, we made the choice to perform only one MAS test before the start of the training protocol. Indeed, for untrained rats, each MAS determination would require a familiarization protocol on the treadmill susceptible to induce adaptation to exercise. So, this would not be consistent for so-called untrained animals to repeat this MAS determination protocol.

In conclusion, this study provides new insights regarding training-induced oxidative capacities and skeletal muscle fibre-dependent adaptations. Regarding transcript results, the HIIT protocol clearly induced an important mitochondrial functional plasticity stimulating aerobic metabolism in *soleus* muscle compared with EDL in Wistar rats. The stimulation at the protein level was only observed in EDL, suggesting muscle-dependent kinetics of transcripts and protein regulatory processes. At the organism level, both HIIT and MICT protocols increased MAS, suggesting an increase in oxidative capacity. The present study could contribute to the improvement of exercise programs adapted to muscle type-dependent responses in order to help to prevent metabolic diseases often associated with mitochondrial dysfunction [49].

## Supporting information

S1 File Original entire blots of PGC-1α, AMPKα and p-AMPKα protein expression in *soleus*. UNTR 7065* was used as an internal control for each blot. GAPDH protein expression was used as a control for each protein of interest (PGC-1α, AMPKα and p-AMPKα). For PGC-1α, AMPKα and p-AMPKα blots, top bands represent proteins of interest, indicated by an arrow. U: UNTR, *n* = 7; M: MICT, *n* = 6; H: HIIT, *n* = 8.
(ZIP)

S2 File Original entire blots of PGC-1α, AMPKα and p-AMPKα protein expression in EDL. UNTR 7065* was used as an internal control for each blot. GAPDH protein expression was used as a control for each protein of interest (PGC-1α, AMPKα and p-AMPKα). For PGC-1α, AMPKα and p-AMPKα blots, top bands represent proteins of interest, indicated by an arrow. U: UNTR, *n* = 6; M: MICT, *n* = 7; H: HIIT, *n* = 8.
(ZIP)
