# Supplementary material for: Moderate intensity continuous versus high intensity interval training: Metabolic responses of slow and fast skeletal muscles in rat
Source: PLoS One. 2023 Oct 4;18(10):e0292225. doi: 10.1371/journal.pone.0292225 (PMC10550171; doi:10.1371/journal.pone.0292225)
Supplement: S1 File — UNTR 7065* was used as an internal control for each blot. GAPDH protein expression was used as a control for each protein of interest (PGC-1α, AMPKα and p-AMPKα). For PGC-1α, AMPKα and p-AMPKα blots, top bands represent proteins of interest, indicated by an arrow. U: UNTR, n = 7; M: MICT, n = 6; H: HIIT, n = 8. (ZIP) [file pone.0292225.s001.zip › S1_Soleus blots.docx]

**Fig S1 A. Original entire blots of PGC-1α protein expression in *soleus***

(kDa)

(kDa)

U 3820	M 7284 H 8476	U 7101	M 8247	H 0000	U 7065*

75

100

150

250

75

100

150

250

XXX	XXX	H 4A	U 7447	M 3773 H 3775	H 7329 U 7065*

(kDa)

(kDa)

75

250

150

100

250

75

150

100

**.**

(kDa)

150

(kDa)

U 3792	M 9715	H 3774	U 3767 M 3799	H 8030	U 7065*

75

250

100

75

100

250

150

(kDa)

(kDa)

U B6	M 3843	H 7633	U 7065*

150

250

100

75

250

150

100

75

**Fig S1 B. Original entire blots of AMPKα protein expression in *soleus***

U 3820 M 7284 H 8476	U 7101	M 8247	H 0000	U 7065*

50 kDa

50 kDa

XXX	XXX	H 4A	U 7447	M 3773	H 3775	H 7329	U 7065*

50 kDa

50 kDa

U 3792	M 9715	H 3774	U 3767 M 3799 H 8030	U 7065*

50 kDa

50 kDa

U B6	M 3843	H 7633	U 7065*

50 kDa

50 kDa

**Fig S1 C. Original entire blots of phospho-AMPKα Thr^172^ protein expression in *soleus***

U 3820	M 7284 H 8476	U 7101	M 8247	H 0000	U 7065*

50 kDa

50 kDa

XXX	XXX	H 4A	U 7447	M 3773 H 3775 H 7329	U 7065*

50 kDa

50 kDa

U 3792	M 9715	H 3774	U 3767	M 3799	H 8030	U 7065*

50 kDa

50 kDa

U B6	M 3843	H 7633	U 7065*

50 kDa

50 kDa

**Fig S1 D. Original entire blots of GAPDH protein expression in *soleus***

(kDa)

U 3820	M 7284	H 8476	U 7101	M 8247	H 0000	U 7065*

(kDa)

37

37

25

25

(kDa)

XXX	XXX	H 4A	U 7447	M 3773 H 3775 H 7329	U 7065*

(kDa)

25

37

37

25

(kDa)

(kDa)

U 3792	M 9715	H 3774	U 3767	M 3799	H 8030	U 7065*

25

37

37

25

U B6	M 3843	H 7633	U 7065*

(kDa)

(kDa)

20

25

37

20

25

37
