# Supplementary material for: Moderate intensity continuous versus high intensity interval training: Metabolic responses of slow and fast skeletal muscles in rat
Source: PLoS One. 2023 Oct 4;18(10):e0292225. doi: 10.1371/journal.pone.0292225 (PMC10550171; doi:10.1371/journal.pone.0292225)
Supplement: S2 File — UNTR 7065* was used as an internal control for each blot. GAPDH protein expression was used as a control for each protein of interest (PGC-1α, AMPKα and p-AMPKα). For PGC-1α, AMPKα and p-AMPKα blots, top bands represent proteins of interest, indicated by an arrow. U: UNTR, n = 6; M: MICT, n = 7; H: HIIT, n = 8. (ZIP) [file pone.0292225.s002.zip › S2_EDL blots.docx]

**Fig S2 A. Original entire blots of PGC-1α protein expression in EDL**

(kDa)

(kDa)

U 3820	M 7284 H 8476	U 7065*	M 8247 H 0000	H 4A

150

250

75

150

100

100

75

250

(kDa)

75

(kDa)

U 7101	M 8740	H 3775	U B6	M 3843	H 7329	H 3774	U 7065*

150

250

100

75

250

150

100

(kDa)

U 7447	M 3773	H 8030	U 3767	M 9715 H 7633	M 3799	U 7065*

100

75

150

250

150

(kDa)

250

100

75

**Fig S2 B. Original entire blots of AMPKα protein expression in EDL**

U 3820	M 7284 H 8476	U 7065* M 8247 H 0000	H 4A

50 kDa

75 kDa

50 kDa

U 7101	M 8740	H 3775	U B6	M 3843	H 7329	H 3774	U 7065*

50 kDa

50 kDa

U 7447	M 3773	H 8030	U 3767	M 9715 H 7633	M 3799	U 7065*

50 kDa

50 kDa

**Fig S2 C. Original entire blots of phospho-AMPKα Thr^172^ protein expression in EDL**

(kDa)

U 3820	M 7284 H 8476	U 7065*	M 8247 H 0000 H 4A

75 kDa

50 kDa

50 kDa

U 7101	M 8740	H 3775	U B6	M 3843	H 7329	H 3774	U 7065*

50 kDa

50 kDa

U 7447	M 3773 H 8030	U 3767	M 9715 H 7633	M 3799	U 7065*

50 kDa

50 kDa

**Fig S2 D. Original entire blots of GAPDH protein expression in EDL**

(kDa)

(kDa)

U 3820	M 7284 H 8476	U 7065*	M 8247 H 0000	H 4A

37

25

37

25

(kDa)

(kDa)

U 7101 M 8740	H 3775	U B6	M 3843	H 7329	H 3774	U 7065*

25

37

37

25

(kDa)

37

(kDa)

U 7447	M 3773	H 8030 U 3767	M 9715 H 7633	M 3799	U 7065*

25

37

25
